# Barriers and motivation for presumptive tuberculosis case referral: qualitative analysis among operators of community medicine outlets in Ghana

**DOI:** 10.1186/s12913-022-08321-7

**Published:** 2022-08-01

**Authors:** M.P Kwabla, C. J. Klett-Tammen, S. Castell

**Affiliations:** 1 Department for Epidemiology, Helmholtz Centre for Infection Research, Brunswick, Germany; 2 Programme Epidemiology, Helmholtz Centre for Infection Research (HZI), HBRS at the Medical School Hannover, Brunswick, Germany; 3 Department of Epidemiology and Biostatistics, School of Public Health, University of Health and Allied Sciences, Ho, Ghana

**Keywords:** Community medicine outlets, Presumptive TB case referral, Ghana

## Abstract

**Background:**

Community medicine outlets (CMOs) are the first point of call for individuals presenting with cough in Ghana. Although operators of CMOs comprising pharmacists and over-the-counter (OTC) medicine sellers largely support the public–private mix strategy which seeks to engage pharmacies in tuberculosis (TB) case detection, a significant proportion is not involved in TB referral services. The study explores the barriers to and motivation for presumptive TB case referral among CMO operators.

**Methods:**

We used open- and close-ended questions nested in a telephone survey which assessed factors associated with presumptive TB case referral among CMO operators (*n* = 465). We interviewed participants using computer assisted telephone interviews and analysed the qualitative data using adjusted Mayring’s structured qualitative content analysis.

**Results:**

Based on participants’ own experiences, non-referral was attributed to negative attitudes of presumed cases (48.2%) and inability to meet the financial demands of referred presumed cases (26.3%). Regarding their perception of barriers to TB referral for their professional colleagues, an assumed lack of TB training (44.5%) and an assumed negative attitude of operators (43.6%) were mentioned. From close-ended questions, most chosen barriers to referral were: the assumption of not having seen a presumptive TB case yet (31.8%), lack of TB training (22.2%) and no monetary motivation for operators (10.5%). Most operators (81.6%) view TB referral services as their social responsibility and feel self-motivated to refer cases in order to control the spread of TB in their communities. Of 152 further comments extracted as recommendations to improve referral, 101 (66.4%) of respondents would only refer with the availability of support systems in the form of TB training and making TB diagnostic testing more accessible.

**Conclusion:**

Operators of CMOs are predominantly self-motivated to refer presumptive TB cases. Barriers to referral might be mitigated by providing more training to operators and specific financial support such as reimbursement of travel costs to presumptive cases.

## Background

According to the World Health Organization (WHO), in 2016, there was a 4.3 million gap between incident and notified tuberculosis (TB) cases globally partly due to under-reporting of detected cases [1]. In Ghana, a key challenge to be addressed to curb the spread of TB is low TB case detection [2]. The 2013 national TB prevalence survey in Ghana among the general population revealed that only 9 out of 202 cases detected via the survey had been diagnosed through the TB surveillance system [3]. Hence, TB case detection in Ghana seems to remain below the 70% target [2, 4]. WHO reconsidered the TB burden in Ghana after the 2013 prevalence survey and the current annual incidence estimate is 143/100,000 population [5].

The Ghana National Tuberculosis Program (NTP) in response to the low case detection adopted the WHO’s public–private sector collaboration strategy that explicitly emphasizes the importance of engaging pharmacies and over-the-counter medicine outlets in TB case detection [6]. Community pharmacies and over-the-counter medicine outlets do not require prescriptions for most of their medications and have short or no waiting time, hence their services are highly frequented by community members including individuals presenting with cough [7]. International studies have shown that operators of community medicine outlets in other countries largely support the public–private mix (PPM) strategy, which also includes referral of individuals with presumptive TB to public health facilities as shown in studies from Pakistan [8, 9], Cambodia [10] and India [11].

Collaboration of the National TB Control programmes (NTP) with pharmacies through referral of presumptive TB to general health care facilities has shown to improve case detection considerably [12–14]. However, there still seems to remain a significant proportion of pharmacies that do not actively participate in TB referral services; e.g., only about half of pharmacies were found to refer presumptive TB to general health care facilities in Vietnam [13]. Besides, not all people referred end up at the designated health facilities (mainly government health facilities with TB diagnostic laboratories) for diagnosis [10]. There remain questions to be answered: If pharmacies are willing to be engaged in the PPM strategy, why are they not all referring most presumptive TB cases who visit their shops? Are they asking the right questions to be able to accurately identify and classify presumptive TB cases? Do they have the tools and the support they need to refer presumptive TB cases?

Lack of a comprehensive understanding of barriers and motivation for presumptive TB referral is a major obstacle to increase effective engagement of pharmacies in TB referral services. Hence, this study examines barriers to and motivation for presumptive TB case referral among pharmacists and over-the-counter (OTC) medicine sellers in the Eastern Region of Ghana.

## Methods

### Study design, participants and data collection

This mainly qualitative content analysis research used free text data from a telephone survey which assessed the associated factors of presumptive TB referral among community medicine outlets operators [15].

The study population consisted of community pharmacists and over-the-counter medicine sellers operating in the Eastern Region of Ghana. We recruited participants between March 2019 and January 2020 via a list obtained from the Eastern Regional TB control programme coordinator. The list contained information on the locations of the shops and their phone contacts. There is an ongoing collaboration between the shop operators and the TB control programme where TB training sessions are held with the operators for TB referral purposes, making them the right audience for this study.

We engaged 465 pharmacists and OTC medicine sellers from the study region in computer assisted telephone interviews to assess what factors hinder and/or encourage presumptive TB referral. One interviewer (MPK) was involved in administering the semi-structured questionnaire via the telephone and entered the responses of interviewees into LimeSurvey. The interviews were conducted mainly in English and a few in local Ghanaian languages all of which were documented in English. The interviews took approximately 18 min on average.

Questions on barriers to referral were asked in three ways. The first part provided a list of barriers for a yes and no responses: lack of training, if non-referral was because operator had not seen a presumptive case in the shop yet, lack of monetary motivation from the TB Programme and forgetfulness of the operator to refer. The second part asked participants for any additional barrier not mentioned in the list (“other reasons” and free text answer). The third part queried participants reasons for non-referral among their colleagues in other pharmacies to measure normative expectations and beliefs of what others think should be done [16]. To assess participants’ motivation for referral, we asked for what factors encourage them to refer (more) presumptive TB cases for laboratory diagnosis (open-ended question). To evaluate the proportion of participants who could accurately identify a presumptive TB case, we asked for how they define a presumed TB case [17, 18] as open-ended question. Finally, we asked for any further comments and used those to extract suggestions on improvement of referral.

### Data analysis procedures

The study includes also quantitative analyses: in order to rank answer categories for identifying the most important topics for further evaluation and recommendations, we reported frequencies as well for the qualitative questions due to the large sample size (n = 465) used. Answers to close-ended questions are given as frequencies. We excluded free text answers on lack of training as a barrier to referral for participants who had indicated training was not a barrier in the closed-ended question before. We used a modified and extended structured qualitative content analysis method with inductive category development according to Mayring’s approach to analyse the open-ended items [19]. This approach allows for a step-by-step formulation of categories based on the content of the study material [20]. Additionally we used a deductive approach to include the use of Ghana’s case detection SOP regarding TB case definition [16]. Two of the authors (MPK and CJK), and a third researcher who had no prior knowledge of the study performed the analysis. MPK and the third researcher independently coded the themes on barriers, motivation and recommendation. CJK and MPK coded the theme on presumptive TB case definition. The last author (SC) supervised the coding process in parts. SC, MPK, CJK and the independent researcher each reviewed the results of the analysis. SC and CJK have had prior experience with qualitative studies.

For each research question, the researchers independently generated categories based on the responses for the coding process [21]. We subjected the categories to several iterations under the inductive process before the researchers agreed on the final version for the codebooks (Table 1). The categories were then applied independently by the researchers on the responses per research question for the coding process. Afterwards, the researchers reviewed the codes assigned to each response for agreements using Cohen’s Kappa [22] interrater reliability testing, to arrive at a consensus (Table 2). Coding of responses was done manually by highlighting codable text and inserting a category as a comment in a word document after which the codes were exported into excel and analysed as relative frequencies of the respective themes. The minimum acceptable level of crude agreement was set at 80%. If this was not attained, the coding process was repeated and consensus was found via a supervised process [22, 23]. Different units in one response were coded to more than one categories, hence, the coded items exceed the number of respondents. We used STATA/IC version 14 to calculate inter-coder reliability coefficients and frequencies.

**Table 1:** Codebook with categories on barriers and motivation for presumptive TB case referral among operators of community medicine outlets in Eastern Region, Ghana

Thematic area	Category	Definition (inclusion/exclusion criteria)	Examples of quotes	N (%)
**Barriers to TB referral among operators themselves, i.e. study participants *N* = 114**	Financial barrier for operators to meet up with the demands of presumptive TB cases ^*^	Lack of money or transport fees	When I refer, I have to give them money before they agree to go Some complain of money for hospital expenses so they do not go to the lab when you refer I pay for their transport fares	30 (26.3)
	Negative attitude of presumptive TB cases	Refusal to go to lab when referred for various reasons	Some refuse to go, they fear hospital When you refer, they refuse to go, they fear to hear the word TB Also because of stigmatization, people may not feel fine when you tell them you suspect TB	55 (48.2)
	Negative attitude of operators themselves	Lack of moral, responsibility and commitment, no regard for law, carelessness, fear of infection	I fear I will get infected by talking to clients I do not feel confortable telling someone I suspect you of TB	12 (10.5)
	Negative attitude of TB programme workers	Complaints by shop operators against TB control programme	No feedback from the people I refer as to whether they were able to go or not Bad relationship between TB program and pharmacy operators	12 (10.5)
	Logistical support barrier	Lack of referral forms, TB kits, license, non-referral due to location	We do not have the forms to refer the people	5 (4.4)
**Assumed barriers to TB referral by professional colleagues *N* = 346**	Lack of training on TB detection for shop operators	Non-referral because of lack of knowledge or no training received	Maybe they have not been trained	154 (44.5)
	Financial barrier for operators to meet up with the demands of referred presumptive TB cases	Lack of money or transport fees	Monetary problem, they don’t have money to accompany the client to the hospital	40 (11.6)
	Negative attitude of presumptive TB cases	Refusal to go to lab when referred for various reasons	Maybe the customer is not giving them the chance to do that They fear they will test positive for HIV	21 (6.1)
	Negative attitude of colleagues	Lack of moral, responsibility and commitment, no regard for law, carelessness, fear of infection	Because of love for money they will sell their drugs than refer the people Maybe they don’t care	151 (43.6)
	Negative attitude of TB programme workers	Complaints by shop operators against TB control programme	Maybe lack of motivation from TB program No proper collaboration between TB programme and OTC	5 (1.4)
	Logistical support barrier	Lack of referral forms, TB kits, license, non-referral due to location	Maybe they do not have sputum containers They do not have the referral forms	14 (4.0)
**Motivation for TB referral *N* = 450**	Social responsibility, self-motivation and disease prevention	Love, empathy, willingness to refer once a case is seen	The hospital is a better place for them to get help so I will refer I know that if I refer and they get diagnosed, they will be treated and become less infectious People with TB suffer a lot and so instead of selling cough syrup, which will not cure, I will refer When I refer, it shows I know my work I cannot cure TB so once I sell the cough syrup and its not working, I have to refer	367 (81.6)
	Availability of support systems for pharmacies and OTCs	Logistical, training, financial, feedback, behaviour of health workers, free treatment	If they train us, then I will be able to suspect and send The people we refer do not have money to go to the hospital so we need help TB test should be done in the communities If you supply us with the sputum containers, I will refer	83 (18.4)
	Positive attitude of presumptive TB cases	All customer related attitudes encouraging operators to refer them	But the customers should open up to me and not to make it too difficult for me […] Once the person is willing to respond, I will refer	10 (2.2)
	Establishment of community TB education and awareness creation	Operators proposed solutions to prevent clients hesitancy	The health directorate should educate the people so that when we refer they will go Awareness creation is needed to prevent stigmatization	8 (1.7)
Definition of presumptive TB case^**^ ***N* = 463**	Coughing for less than 2 weeks	Coughing for any number of days less than 14 days	Person is coughing for a week Coughing for more than a week	59 (12.7)
	Coughing for 2 weeks or more	Cough lasting any duration of 14 days or more	Coughing for 2 weeks When the person is coughing for more than 2 weeks	73 (15.8)
	Coughing over a period of more than 2 weeks	Cough lasting over 2 weeks or more	Coughing more than 3 months Somebody coughing more than a month	54 (11.7)
	Cough without specific duration	Cough of any duration without time frame	When customer complains of persistent cough Someone coughing for a long time	269 (58.1)
	Night sweat	Any mention of night sweat	Coughing with night sweats	37 (8.0)
	Fever	Any mention of fever	Cough with fever	10 (2.2)
	Chest pain	Any mention of chest pains	Productive cough, chest pains	24 (5.2)
	Weight loss	Any mention of weight loss	Coughing frequently and growing lean	59 (12.7)
	Other TB related symptoms	Blood in sputum, weakness, fatigue, loss of appetite, sputum	Persistent dry cough for more than a month with blood Lack of appetite	173 (37.3)
	Cough that is not going away with cough medication	Cough that is not relieved with medicine	If after taking cough mixture, they still don't get better	162 (35.0)
	Unrelated symptoms	Any belief regarding behavioural characteristics or medical symptoms not fitting into any of the other categories	Drinking alcohol Eating with people in the night Body changes Vomiting Headaches breathlessness	61 (13.2)
**Recommen-dation to improve TB referral *N* = 126**	Availability of support systems for pharmacies and OTCs	Logistic, training, monetary, feedback, materials, supplies, facilities, labs	I will suggest we are given the containers so we can help We should be supported with transportation money for those we refer TB programme should give training to OTC medicine sellers so we don’t just sell cough syrup but also refer There should be a lab in the health centres within reach for the people	101 (80.2)
	TB awareness creation and health education	Proposed solutions to deal with refusal of referred clients to visit the health facility	There should be TB awareness so that when we refer they will go to the hospital Mostly because of high illiteracy rate here, when you refer, they refuse to go	31 (24.6)
	Empowerment and regulation of pharmacies and OTCs	Ease of restrictions and permission to sell drugs, monitoring of license	The pharmacy council restricts us from selling some pain killers and we are not making much profit as a result We need permission to sell class A drugs especially for those of us in the villages Illegal chemical sellers should be regulated, they go round sellings drugs they are not allowed to sell	20 (13.2)

^*^ Although we asked for barriers based on lack of training and financial support by the TB programme in the closed-ended question, some interviewees mentioned closely related barriers in their free text answers on further barriers which are mentioned here

^**^ Duration of cough was treated separately from the naming of the symptom to investigate the proportion of respondents that named symptoms correctly as well as the duration of cough

**Table 2:** Inter-coder reliability of two raters using Cohen’s Kappa

Research question/Main theme	No. of items	Crude agreement (%, n)	Kappa coefficient ( ^*^CI)
Barriers to TB referral among participants	120	84.2 (101)	0.78 (0.70–0.87)
Barriers to TB referral among colleagues	385	85.0 (327)	0.79 (0.74–0.83)
Motivation for TB referral	468	92.5 (433)	0.81 (0.78–0.87)
Knowledge of presumptive TB case definition	981	95.3 (935)	0.94 (0.93–0.96)
Recommendation to improve referral	152	92.8 (141)	0.86 (0.79–0.94)

^*^ *CI* confidence interval

## Results

### Background characteristics of operators of community medicine outlets and responses

Of all 465 participants analysed, 341 (73.3%) were males, 349 (75.1%) had a secondary level education and were aged between 20–86 years (15). For the theme on barriers to TB referral among participants and their colleagues, the number of respondents were 114 and 346 respectively and the responses were grouped into six categories or sub-themes. On motivation for referral, there were 450 respondents who provided responses on what prompts them to refer presumptive TB cases who visit their shops with four sub-themes. Knowledge of presumptive TB case definition had 463 respondents with eleven sub-themes while that on recommendation had 126 respondents with responses grouped into three categories (Table 1).

### Knowledge of presumptive TB case definition among participants

We asked the operators about how they define a presumptive TB case in their shop and we grouped the definitions into eleven sub-themes; responses of the 463 respondents resulted in 981 quotes. The basis of defining a presumptive TB case according to Ghana’s SOPs (18) was for a participant to know the important symptoms suggestive of TB and/or duration of cough. Of the 463 respondents, more than half, 269 (58.1%) could only define a presumptive TB case as one who is coughing persistently for a long time without any specific duration, 73 (15.8%) knew the correct duration of cough of two weeks or more according to Ghana’s SOP on TB case definition and 173 ((37.3%) could mention other TB related symptoms such as blood in sputum and loss of appetite. Of 463 participants, 24.6% (*n* = 114) mentioned at least one of the cardinal symptoms of TB including fever 10 (2.2%), night sweat 37 (8.0%), weight loss 59 (12.7%) and chest pain 24 (5.2%) with 15 persons (3.2%) stating at least two of them. In addition to the classical TB symptoms to check for in presumptive cases, 162 (35.0%) of participants talked about their own system of referring only if the customer had come to request for cough syrup more than once: *“When I see a customer for the 1st time complaining of cough, I give them cough syrup, if I see the same person the 2nd time, I ask them to go to the hospital* (*Female, shop assistant)*.“ Likewise, 13.2% (61/463) mentioned symptoms that are officially unrelated to TB such as vomiting and shiny eyes.

### Barriers to referral of presumptive TB among operators of community medicine outlets stratified by referral status

Based on four pre-defined answers categories, among those who reported having ever referred, 19.3% (62/321) indicated a lack of TB training as a major barrier that would have prevented them from making a referral, followed by a lack of monetary motivation from the TB control program (8.4%, 27/321) while 7.2% (23/321) attributed non-referral to the assumption that no case of presumptive TB is seen in their shops, 5.9% (19/320) agreed that they forgot to refer. Among those who stated having never referred a presumptive TB case, 28.5% (41/144) attributed their non-referral to a lack of training on TB detection, 15.3% (22/144) was due to lack of monetary motivation from the TB programme, 86.8% (125/144) was because they assumed they had not seen a presumptive TB case at their shop yet and 2.1% (3/144) reported to have forgotten to refer.

### Additional barriers to referral of presumptive TB cases among participants

Of the 49 participants who agreed with lack of monetary motivation in the pre-defined question, 7 (14.3%) gave redundant free text linking non-referral to when no monetary motivation was available. In addition, of 414 who disagreed with lack of monetary motivation by the TB programme, 23 (5.5%) gave additional free text on clients refusal to go when referred due to transport cost warranting operators to use their own money to support clients.

Of the 465 participants who responded to the pre-defined answer category question, 114 mentioned additional barriers when asked for any other reasons which emerged in six thematic areas with 120 quotes. Fifty-five interviewees (48.2%) attributed non-referral to negative attitudes of presumed cases. They said: *“The people don't like it when you tell them you are suspecting TB so sometimes its difficult to tell them.” (Female, shop owner).*; “*When you refer, they refuse to go, they fear to hear the word TB.” (Male, shop owner).; “Some refuse to go, they fear hospital.” (Male, shop owner).* Participants also mentioned a lack of financial support (30/114, 26.3%) such as operators having to use their own money to cater for transportation cost of clients: “*When I refer I have to give them money before they agree to go to the hospital”. (Male, shop owner).* Ten and a half percent (12/114) of participants would not refer a presumptive TB case due to reasons such as feeling uncomfortable to tell customers they suspect them of TB and fear of losing customers to other shops. Examples of some quotes are: *“I do not feel confortable telling someone I suspect TB.’’ (Male, shop owner).; “You may loose customers when you keep referring customers.’’ (Male, shop owner).* Another 10.5% (12/114) of participants mentioned the lack of feedback from the TB workers and clients complaints of long waiting time at health facilities as barriers to presumptive TB referral: *“The health coordinators are not coorporating with us, they do not give us feedback whether the people we refer have gone to them or not, the patients also refuse to go because of long queues’’. (Male, shop owner).* Unavailability of logistical support including the lack of TB referral forms and sputum containers for collection of samples is a barrier to referral among 4.4% of participants (5/114). Some participants put it this way: *“We d*o *not have referral forms and so we only do it verbally which is not good.” (Male, shop owner).; ‘‘We were told we would be given sputum containers but not yet given.’’ (Male, shop owner).*

### Participants’ perception on barriers to presumptive TB referral among professional colleagues in other community medicine outlets

A total of 346 participants provided free text on perceive barriers to referral among their professional colleagues which emerged in six thematic areas with 385 quotes details of which are described as follows.

### Lack of training on TB detection for shop operators

An assumed barrier to TB referral mentioned by 44.5% (154/346) of participants is a lack of TB training hence, they lack the prerequisite knowledge about asking the right questions, identify and refer. In the words of a participant when asked what they perceive as a barrier to referral among their colleagues: “*Maybe lack of training so they lack knowledge on how to identify TB.” (Male, shop owner).* Participants also expressed worry that the training sessions are not on a regular basis and mainly focused on the shop owners leaving their assistants untrained. Their responses here were: “*The training was done for selected people so maybe some are not trained.’’ (Male, shop owner).; “Sometimes the assistant there may not be knowledgeable enough to do it.” (Male, shop owner).; “No follow up after last training, after learning something for a long time, you forget so frequent trainings will be fine.’’ (Male, shop owner).; ‘’They should organize training for us. New ones are coming in who need to be trained.’’ (Male, shop owner).*

### Lack of financial support to meet demands of referred TB clients

According to 11.6% (40/346) of participants, referred presumed cases refused to visit the designated health facilities until operators agree to pay for their transport cost, hence colleague operators would prefer not to refer at all than to refer a client who refuses to visit the designated facility. To them, a prerequisite for referral is for the operator to be willing to meet the financial demands of referred cases and since they do not receive any financial support from the TB program, this discourages them from referring presumed cases. Examples of some quotes on this theme are: “M*aybe when they refer, the people don't go because of transport fares.” (Male, shop owner).;* “*Monetary problem, they don’t have money to accompany the client to the hospital.” (Female, shop owner).; “Lack of resource support since they have to bare the cost of client transport.’’ (Male, shop owner).*

### Negative attitude of presumptive TB cases

Of all barriers to referral, 6.1% (21/346) of participants assumed their colleagues would most likely not refer a customer who showed up in their shops exhibiting unfriendly attitude and not willing to be spoken to. Related quotes on this theme include: *“Maybe the customer is not giving them the chance to do that.” (Male, shop owner).; “Maybe the willingness of the people referred to go to the hospital is not there and that put them [i.e. the colleagues] off.” Female, shop owner).*

### Negative attitude of pharmacists and OTC medicine sellers

When participants were asked about barriers to TB referral among their colleagues in other OTCs, 43.6% (151/346) mentioned they thought their colleagues were just not caring for the welfare of the people, greed and profit maximization leading to many wanting to only sell their medicines without the consideration to refer the cases to the health facilities where they can be well managed. They also mentioned fear of infection in the course of talking to the clients*: “Because of love for money they will sell their drugs than refer the people.’’ (Male, shop assistant).; “Maybe they don’t care or they don’t see TB as a bad disease.” (Male, shop owner).; “Wickedness.” (Male, shop owner).; ‘’Maybe they are only concerned about the profit of selling their medicines or they don't have time.’’ (Female, shop owner).; Maybe they are afraid they will get infected by talking to the customers.” (Male, shop owner).*

### Negative attitude and behavior of the TB programme workers

A few (5/346, 1.4%) of the participants mentioned negative attitude of the TB workers, i.e. health workers at the hospitals, as a barrier to referral among their colleagues. In a quote, a participant said: *“We do not have permission to follow up on the hospital to know if the people we referred went to the hospital so that could be the reason some don't want to refer.” (Male, shop owner).*

### Lack of logistical support for pharmacies and OTCs

A total of 14 (4.0%) participants on behalf of their colleagues in other OTCs indicated that non-referral could be linked to unavailability of logistical support e.g. the TB referral forms and the lack of TB labs that are within reach to presumptive TB cases. A few of them on this theme said: “*Maybe they do not have sputum containers.” (Male, shop owner).; “Maybe no nearby laboratory.” (Male, shop owner).*

### Motivation for presumptive TB referral among participants

A total of 450 participants provided free text response on motivation for TB referral which were categorized into four sub-themes with 468 quotes details of which are described as follows:

### Social responsibility, self-motivation and disease prevention

Of all factors that motivated referral, 81.6% (367/450) of operators view TB referral services as their social responsibility and that they are mandated by the license given them to operate to rid their communities of diseases. They said: “*We are given the license so we can serve the community so once I see one (a suspected TB case), I will refer,” (Male, shop assistant).; “That's my profession and I'm in to help my community.’’ (Male, shop owner).;* Apart from the belief that failure to refer would result in spread of TB among close relatives, they also believe they do not have the cure for TB and, hence, the need to refer presumed cases to the public health facilities where they can be diagnosed and treated. Selected quotes on this theme include: *“TB is infectious even to myself and the families so I can't delay in referring,” (Male, shop owner).; “My own personal motivation, if I do not refer, I may be affected.” (Female, shop owner).; “TB treatment is free. My medicine cannot cure so I have to refer.” (Male, shop owner).*

### Availability of support systems for pharmacies and OTCs

For 18.4% (83/450) of participants, their motivation for referral is tied to receiving support in the form of training on TB detection, feedback on referred cases, financial support and regular visit by the TB control workers and making TB diagnostic testing services available within reach to the people in the communities. Some participants in relation to this theme said: *“If they train us, then I will be able to suspect and send.” (Male, shop owner).;”When I refer them and I get a feedback that they are doing well, it makes me happy.” (Male, shop owner).; “Financial assistance from the TB program and constant visit.” (Male, shop owner).; “Sometime you refer and they refuse to go. If the TB people in my district will come and do the test in my community, I will like it.” (Male, shop owner).*

### Positive attitude of persons with presumptive TB

Motivation for referral for 2.2% of participants (10/450) include receiving customers who are well behaved with an attitude devoid of difficulties in offering them suggestions and who are willing to visit the designated health facility when referred. *“The customers should open up to me and not to make it too difficult for me. Because of stigma, most of them will not like you to suspect them of TB.” (Male, shop owner).; “Once the person is willing to respond, I will refer. Some of them refuse to go to the hospital.” (Male, shop owner).*

### Establishment of community TB education and awareness creation

Some shop operators (8/450, 1.7%) believe their customers lack basic TB knowledge resulting in resistance to visit the health facilities when referred. Hence, they would be motivated to refer if the TB control programme educates the public using various mediums of communications. Examples of quotes on this include: ***“****The health directorate should educate the people so that when we refer, they will go.” (Male, shop owner).; “We need to advertise with TB flyers or posters. Awareness creation is needed to prevent stigmatization.” (Male, shop owner).*

### Recommendation to improve presumptive TB referral among participants

We asked participants for optional comments in the form of questions and suggestions to improve referral of presumptive TB cases and this emerged in three sub-themes; 126 participants answered resulting in 152 quotes.

### Availability of support systems and training for pharmacies and OTCs

Of all recommendations given, 80.2% (101/126) of participants suggested providing operators with regular training to keep them up to date on issues relating to TB and financial support to meet the demands of referred presumed cases. Quotes here include: *“TB is contagiuos so we need some training. Its been long since we receive any training.” (Male, shop owner).; “When you refer, they don't go and we use our own money so we need support in that.” (Male, shop owner).*

### TB awareness creation and health education

Similar to motivation factors, 24.6% of participants (31/126) recommend providing TB education to bridge the TB knowledge gap among the general public. *“TB programme should use the information van to create awareness. Most people don’t know TB diagnosis and treatment is free.” (Female, shop owner).*

### Empowerment and regulation of pharmacies

Of concern to 13.2% of OTC medicine sellers (20/126) is for the pharmacy council to regulate illegal drug operators and at the same time ease, the restrictions for those with licenses to have more dispensing options. Examples of quotes here are: *‘’The pharmacy council restricts us from selling some pain killers and we are not making much profit as a result.’’ (Male, shop owner).; ‘’There's so much difficulty getting license from pharmacy council so this has to be looked at.There are many quack doctors who go round selling medicine in the communities without licence. Those of us who do not want to behave like that and apply, they should try and give it to us.’’ (Male, shop owner).; ‘’Some people do not have the licence but they are going round selling medicines, the pharmacy council should check that.” (Male, shop owner).*

## Discussion

PPM collaboration with pharmacies require the shop operators to refer every single presumptive TB case that visits their shops. Lack of TB training, financial and logistical support, negative attitudes of presumed cases, operators themselves and the TB programme workers were identified as major barriers to such presumptive TB case referral. Over 28% of participants who have never made presumptive TB referral attributed it to a lack of training. It seems also clear from the responses that the training has been erratic as many expressed worries that the new operators coming in have not been trained. The lack of TB training could partly explain why over 30% of our participants attributed their non-referral to not having seen a presumptive TB case yet, which is reflected by the high number of respondents who could not give the right case definition. Although staff turnover was highlighted as creating a knowledge gap in TB case detection in a study on barriers to TB case finding in Ghana [24], this did not come up as a barrier to referral in our study. Of the respondents, 10.5% and 26.3% in the pre-defined and free text responses respectively attributed non-referral to a lack of financial support by the TB programme leading to some operators reporting to have to bear the cost of transportation for the customers they referred. Costs associated with transportation hindered access to health facilities e.g. in Nepal as well [25]. In a study in Cambodia, support to pharmacy staff with the tools to work with and a system to ensure follow up on referred patients encouraged referral among the participants [10].

For 48.2% of participants, non-referral was attributed to a negative attitude put up by the customers who visit their shops, partly due to their refusal to report to the designated health facility and also because they were not open enough to allow the shop operators talk to them. Some participants also think they may lose customers to other pharmacies as it was also seen in Vietnam [26]. Some presumed TB clients are not literate, they lack sensitization on TB and were unaware of availability of cure for TB as was also seen in India [27] and, hence, referral to a hospital meant a death sentence. Almost half (48.2%) of our respondents are of the opinion that long waiting time at the hospital and stigmatization of TB patients discourages the presumptive TB cases from visiting the health facilities when referred similar to a findings in India [27] hence, the providers often do not bother referring them for sputum test. In a study in Portugal, stigmatization of TB patients at directly observed treatment (DOTs) centres led to poor treatment outcomes compared to those receiving treatment from pharmacies [28]. Therefore, efforts to address negative attitude of clients to improve TB referral should include tackling stigmatization of patients through TB education in societies. Non-referral by 43.6% of colleagues of the participants was assumed to be linked to their own negative attitudes including fear of contracting TB during conversation with customers, lack of skills on how to start such conversations with customers and their love for money and profit maximization motive which propelled them to sell their drugs without the consideration to refer. This is also seen in other countries: Pharmacy staff in Cambodia fear they or their families might contract infections during patient counselling, hence perceive it as a barrier to referral [29]. Likewise in Viet Nam, 7.8% of pharmacy staff felt uncomfortable telling their clients they suspect TB [26] partly because the capacity of professional staff was considered too low to be able to accurately diagnose TB [30]. Pharmacy staff in our study mentioned a negative attitude of the TB workers including a feedback loop on status of cases referred and lack of referral forms making their referral verbal and undocumented as a barrier to TB referral. Similarly, patients’ refusal to receive health care was attributed to health care workers attitude in India [31]. However in India, incentive support for pharmacy providers and monthly SMS on status of referred clients increased the number of referrals [11].

Motivation for presumptive TB referral comes in the form of participants own intrinsic motivation and the quest to rid their communities of diseases, receiving training, financial and feedback support, a positive attitude of presumed cases and provision of TB education in the general population. Participants believed part of the reasons for which they are given the license to operate was so that they can contribute to preventing spread of diseases in their communities. They know they do not sell anti-TB medications and hence the best remedy is to identify and refer clients to where they can be diagnosed and treated. Their motivation was also to ensure they or their families do not fall victims to the TB disease. This could explain why in studies in Pakistan [8] and Cambodia [10], most pharmacy staff show no hesitation in signing to participate in the PPM contract.

Supports in the form of training, provision of TB referral forms and sputum containers, feedback on referred cases and financial support are key motivation factors for TB referral in our study. Many studies with success stories on referral by pharmacies emphasized the need to provide training for pharmacy staff [29] and support services including incentives as avenues for sustenance [8, 32]. Participants are motivated when they received clients who are open to the suggestions of pharmacists and who have some basic knowledge on TB. Lack of TB knowledge was also identified as a barrier for delay in seeking TB diagnostic services in China [33].

Only 15.8% of the participants actively reported the correct definition of a presumptive case with duration of cough of two weeks or more. The conflicting information on duration of cough for a presumed case stated in Ghana’s SOP and that of the WHO document [17, 18] could be one reason for this. Between 2.2% and 12.7% reported at least one cardinal symptom. Over one-third (35.0%) of participants used their own system of referring if the customer had come to request for cough medication more than once. A few (13.2%) even mentioned symptoms that are officially unrelated to TB such as eating with people in the night. This could be attributed to the lack of (quality of) training. In contrast, over two-thirds (77%) of pharmacy staff in Peru had adequate TB knowledge and would refer clients with history of cough of 2 weeks or more; at the same time they had myths about transmission of TB [34] as was seen in our study.

Further comments extracted as recommendations to improve referral services were similar to motivation factors including availability of support systems (training and financial), empowerment of OTCs (removing restrictions so that they can have more dispensing options) and creating TB awareness in the general population. In our study, policies by the Ghana Pharmacy Council allowing operators to have a wide range of dispensing options would motivate referral. This agrees with the finding that in most developing counties where trained pharmacists are scarce, there should be exceptions for over-the-counter medicine sellers to sell some prescription based medicines in a bid to extend reach of essential drugs to their communities [35].

The limitation of this study was the inability to audio record the interviews, hence some details may have been missed during the manual capturing process. The strength of the study is its coverage in terms of rather large sample size.

## Conclusion

The major barriers to TB referral were lack of (potentially adequate) TB training for operators, lack of financial support to meet the demands of presumed cases and a negative attitude among operators and presumed cases. Operators of community medicine outlet feel self-motivated to refer presumptive TB cases, however, correct knowledge of e.g. case definition is missing. To enhance this behavior of operators, we recommend that the TB control program should consider intensifying TB education in the general population, provide TB trainings and consider revising the training content and provide financial support in order to sustain the gains made in public–private partnership with pharmacies.

## Data Availability

The datasets used and analysed during the current study are available from the corresponding author on reasonable request within 10 years of the study. Request will be assessed by the institutional data protection officer.

## Abbreviations

CMOs: Community medicine outlets
TB: Tuberculosis
WHO: World Health Organization
NTP: National Tuberculosis Program
PPM: Public–Private Mix
OTC: Over-the-counter
DOTs: Directly observed treatment
